# Loss of Cellular Sialidases Does Not Affect the Sialylation Status of the Prion Protein but Increases the Amounts of Its Proteolytic Fragment C1

**DOI:** 10.1371/journal.pone.0143218

**Published:** 2015-11-16

**Authors:** Elizaveta Katorcha, Nina Klimova, Natallia Makarava, Regina Savtchenko, Xuefang Pan, Ida Annunziata, Kohta Takahashi, Taeko Miyagi, Alexey V. Pshezhetsky, Alessandra d’Azzo, Ilia V. Baskakov

**Affiliations:** 1 Center for Biomedical Engineering and Technology, University of Maryland School of Medicine, Baltimore, Maryland, United States of America; 2 Department of Anatomy and Neurobiology, University of Maryland School of Medicine, Baltimore, Maryland, United States of America; 3 Division of Medical Genetics, Sainte-Justine University Hospital Research Center, University of Montreal, Montreal, QC, Canada; 4 Department of Genetics, St Jude Children’s Research Hospital, Memphis, Tennessee, United States of America; 5 Institute of Molecular Biomembrane and Glycobiology, Tohoku Pharmaceutical University, Sendai, Miyagi, Japan; IRCCS - Mario Negri Institute for Pharmacological Research, ITALY

## Abstract

The central molecular event underlying prion diseases involves conformational change of the cellular form of the prion protein (PrP^C^), which is a sialoglycoprotein, into the disease-associated, transmissible form denoted PrP^Sc^. Recent studies revealed a correlation between the sialylation status of PrP^Sc^ and incubation time to disease and introduced a new hypothesis that progression of prion diseases could be controlled or reversed by altering the sialylation level of PrP^C^. Of the four known mammalian sialidases, the enzymes that cleave off sialic acid residues, only NEU1, NEU3 and NEU4 are expressed in the brain. To test whether cellular sialidases control the steady-state sialylation level of PrP^C^ and to identify the putative sialidase responsible for desialylating PrP^C^, we analyzed brain-derived PrP^C^ from knockout mice deficient in *Neu1*, *Neu3*, *Neu4*, or from *Neu3/Neu4* double knockouts. Surprisingly, no differences in the sialylation of PrP^C^ or its proteolytic product C1 were noticed in any of the knockout mice tested as compared to the age-matched controls. However, significantly higher amounts of the C1 fragment relative to full-length PrP^C^ were detected in the brains of *Neu1* knockout mice as compared to WT mice or to the other knockout mice. Additional experiments revealed that in neuroblastoma cell line the sialylation pattern of C1 could be changed by an inhibitor of sialylatransferases. In summary, this study suggests that targeting cellular sialidases is apparently not the correct strategy for altering the sialylation levels of PrP^C^, whereas modulating the activity of sialylatransferases might offer a more promising approach. Our findings also suggest that catabolism of PrP^C^ involves its **α-**cleavage followed by desialylation of the resulting C1 fragments by NEU1 and consequent fast degradation of the desialylated products.

## Introduction

Prion diseases or transmissible spongiform encephalopathies are fatal neurodegenerative disorders that can be sporadic, inheritable or transmissible in origin [1]. The central molecular event underlying prion diseases involves conformational change of the cellular form of the prion protein denoted PrP^C^ into the disease-associated, transmissible form denoted PrP^Sc^ [2]. Upon expression, PrP^C^ undergoes posttranslational modifications that involve attachment of up to two N-linked glycans to residues Asn-180 and Asn-196 and a glycosylinositol phospholipid anchor (GPI) to the C-terminal residue Ser-230 (residue numbers are given for mouse PrP^C^) [3–5]. Each of the two N-linked glycans can carry up to five terminal sialic acid residues that are linked to the galactose residues at the C-6 or C-3 positions [6, 7]. In addition to sialylation of N-linked glycans, a single sialic acid was also found on the GPI anchor of PrP^C^ [3]. Variation in structure and composition of N-linked glycans gives rise to more than 400 different PrP^C^ glycoforms [6]. Considering heterogeneity in sialylation level of individual glycans and GPI, each PrP^C^ molecule could contain from 0 to 11 sialic acid residues.

The sialylation level of PrP^C^ and brain-derived PrP^Sc^ was found to be very similar, suggesting that sialoforms of PrP^C^ that are sialylated less or more than the statistical average for PrP^C^ were recruited into PrP^Sc^ proportionally to their relative presence in a mammalian brain [7]. However, in prions generated *in vitro* via Protein Misfolding Cyclic Amplification with beads (PMCAb), hyposialylated sialoforms (sialylated less than the statistical average for PrP^C^) were found to be overrepresented at the expense of hypersialylated sialoforms [8]. As a result, the statistical average sialylation level of PMCAb-derived PrP^Sc^ is considerably less than that of brain-derived PrP^Sc^. Surprisingly, in animals inoculated intracerebrally with PMCAb-derived PrP^Sc^ the incubation time to disease was longer than that in control groups [8]. Moreover, animals inoculated with PrP^Sc^ produced from enzymatically desialylated PrP^C^ lacked any clinical signs of prion disease or prion-associated pathology [8]. The correlation between sialylation status of PrP^Sc^ and incubation time to disease gave rise to a new hypothesis that progression of prion diseases could be controlled or reversed by reducing the sialylation level of PrP^C^, the substrate of PrP^Sc^ [8].

Sialylation of glycoproteins is controlled by two groups of enzymes: sialyltransferases and neuraminidases or sialidases [9, 10]. After biosynthesis from monosaccharides in the cytoplasm and subsequent activation in the nucleus, sialic acids are attached to the termini of glycoproteins or glycolipids by sialyltransferases in the Golgi [11]. During recycling or degradation of cell surface molecules, sialic acid residues can be released from glycoconjugates in lysosomes by sialidases. Sialic acid residues can then be recycled or degraded. In addition to lysosomes, desialylation of glycoproteins can also take place at the plasma membrane, a mechanism that is involved in regulating biological activity of cell surface proteins and cell signaling pathways [12].

To test whether the sialylation status of PrP^C^ can be modulated, sialidases were chosen as the first potential targets in the current study. Only four sialidases are expressed in mammals, versus twenty sialyltransferases, and several sialidase knockout mouse models are available. Of the four mammalian sialidases or neuraminidases (NEUs), NEU1 is known to localize to the lysosomes and cell surface, NEU2 is found in the cytoplasm, NEU3 is at the plasma membrane, and NEU4 is associated with mitochondria, lysosomes and ER, but can also be recruited to the cell surface (Table 1) [9, 12, 13]. While NEU1 appears to be involved in regulating numerous signaling pathways, lysosomal catabolism of glycoproteins and oligosaccharides is among its main biological functions [9, 12]. Two neurodegenerative lysosomal storage disorders, sialidosis and galactosialidosis are attributed to deficiency of NEU1 [14–16]. NEU3 and NEU4 are both involved in neuronal differentiation and neurite formation, although they act in opposite directions [17–19]. NEU2 is expressed in muscle tissues and implicated in myoblast differentiation [20]. NEU2 is also expressed in a mouse brain (but not in a human brain) [21], although it is primarily localized in the cytosol (Table 1). For the above reasons, three out of four mammalian neuraminidases, NEU1, NEU3 and NEU4, were examined in the current studies.

**Table 1:** Subcellular localization and substrate specificity of mammalian sialidases.

	Localization	Substrate specificity	Hydrolysis rate ^a^	Expression level relative to NEU1^b^
NEU1	LysosomesPlasma membrane	OligosaccharidesGlycoproteins	α2–3 = α2–6	1
NEU2	Cytosol	OligosaccharidesGlycoproteinsGangliosides	α2–3	1/5,000^th^ of NEU1
NEU3	Plasma membraneRafts Endosomes	GangliosidesOligosaccharides Glycoproteins	α2–3 > α2–6	~1/5^th^ of NEU1
NEU4	LysosomesMitochondriaERCell surface	OligosaccharidesGlycoproteinsGangliosides	α2–3 > α2–6	~1/3^d^ of NEU1

^a^ The hydrolysis rate was assessed for natural substrates [9] and for synthetic substrates using recombinant NEU2, NEU3 and NEU4 and endogenous mouse kidney NEU1 [32]

^b^ Expression level in human brain was assessed by a quantitative real time PCR [67].

There is a considerable overlap between the range of biological activities observed for PrP^C^ and those of NEUs, suggesting that PrP^C^ physiological function could depend on its sialylation. In human embryonic stem cells, PrP^C^ was found to control the switch between self-renewal and differentiation and to promote differentiation of stem cells toward neuron-, oligodendrocyte-, and astrocyte-committed lineages [22, 23]. PrP^C^ was also found to induce polarization, synapse development and neuritogenesis in embryonic neuron cultures [24, 25]. During development PrP^C^ was detected on the growing axons, whereas in the adult brain it was localized along fiber bundles that contain elongating axons [26, 27]. Concomitantly, NEU3 was found to be up-regulated during neuronal differentiation and its expression accelerated neurite formation [17, 18], whereas NEU4 was shown to be down-regulated during differentiation of neuronal cells and its overexpression suppressed neurite formation [19].

To test whether cellular NEUs determine steady-state levels of PrP^C^ sialylation and to identify the enzyme responsible for desialylating PrP^C^, we analyzed brain-derived PrP^C^ from knockout mice deficient in *Neu1*, *Neu3*, *Neu4*, or from *Neu3/Neu4* double knockout. Surprisingly, no differences in sialylation of PrP^C^ or its proteolytic product C1 were found in any knockout mice tested in comparison to the age-matched controls. C1 is generated as a result of proteolytic cleavage of full-length PrP^C^ known as α-cleavage and consists of the C-terminal fragment 111/112-230 [28, 29]. However, significantly higher amounts of the C1 fragment relative to full-length PrP^C^ were found in the brains of *Neu1* knockout mice as compared to wild type controls or *Neu3*, *Neu4* and *Neu3/Neu4* knockout mice. Additional experiments revealed that in cultured neuroblastoma cell line the sialylation pattern of C1 changed by treatment with an inhibitor of sialylatransferases. In summary, the current study indicates that targeting cellular sialidases may not be an effective strategy for altering the steady-state sialylation levels of full-length PrP^C^, whereas manipulating the activity of sialylatransferases may represent a more promising approach. Our results also suggest that catabolism of PrP^C^ involves its α-cleavage followed by desialylation of the resulting C1 fragments by NEU1 and consequent very fast degradation of desialylated C1.

## Materials and Methods

### Brain materials

Three male *Neu1* knockout brains and three male age-matched controls were collected from 4-month old mice of FVB background; one male *Neu1* knockout brain and corresponding age-matched control were collected from 21-weeks old mice of C57Bl6 background [30]. Brains from all *Neu1* knockout mice and corresponding control mice were provided by Dr. D’Azzo (St. Jude Children’s Research Hospital, Memphis, Tennessee); the animal protocol was approved by the Animal Care and Use Committee of St. Jude Children’s Research Hospital, Memphis, Tennessee (Permit Number: 235-100293-03/14). Three brains were used for each of following groups: (i) *Neu3* knockout [21], (ii) *Neu4* knockout [31] and (iii) double knockout *Neu3/Neu4* [32]. All of these mice were male of C57Bl6 background and sacrificed at three months; the respective controls were three male C57Bl6 mouse brains sacrificed at three months. Brains from *Neu3*, *Neu4 and Neu3/Neu4* knockout mice and corresponding control mice were provided by Dr. Pshezhetsky (Sainte-Justine University Hospital Research Center, Montréal, Canada); the animal protocol was approved by the Animal Care and Use Committee of Sainte-Justine University Hospital Research Center, Montréal, Canada (Permit Number: 533).

### Preparation of brain materials for gel electrophoresis and 2D analysis

Mice were euthanized by sodium pentobarbital and perfused with 20 mL PBS, 5mM EDTA (pH 7.4), collected aseptically and stored at −80°C for future analysis. 10% (wt/vol) brain homogenates were prepared as described previously [33]. Briefly, homogenization was performed on ice in PBS, pH 7.4, using glass/Teflon tissue grinders attached to a cordless 12V compact drill (Ryobi). The brains were ground at low speed until homogeneous and stored at -80°C in 1 ml aliquots.

For 2D, 200 μl of 10% brain homogenate was diluted 2-fold in PBS buffer supplied with proteinase inhibitors (cat# 1836145, Roche) and sonicated for 30 seconds in a water bath at 37°C. The sample was subsequently centrifuged at 16,000 g at 4°C for 30 minutes, the supernatant was discarded, and the pellet dissolved in 100 μl 1% Triton X-100 in PBS. 18 μl of brain material was mixed with 6 μl 4x LDS sample loading buffer (cat # NP0007, Life Technologies, Carlsbad, CA), incubated for 10 minutes at 95°C and subsequently used for SDS-PAGE or 2D gel electrophoresis.

### Culturing of N2a cells and preparation for 2D analysis

N2a cells were kindly provided by Dr. Ghaemmaghami [34]. N2a cells were cultured at 37°C, 5% CO_2_ in Minimum Essential Medium (MEM, cat # 10-010-CV, Corning, Corning, NY) supplemented with 10% fetal bovine serum (FBS, cat # 10437, Life Technologies), antibiotics (1% v/v of penicillin/streptomycin, cat # 15140, Life Technologies), and 1% GlutaMAX. Cells were plated at 70% confluency. The sialic acid precursors N-Acetyl-D-mannosamine (ManNAc, cat # A8176, Sigma-Alidrich, St Louis, MO), tetraacetylated N-Azidoacetylmannosamine (Ac4ManNAz, cat # 88905, Thermo Scientific) or sialidase inhibitor 2-deoxy-2,3-didehydro-d-N-acetylneuraminic acid (DANA, cat # D9050, Sigma-Aldrich, St Louis, MO) were added at concentrations of 10 mM, 4 mM or 5 mM, respectively. After incubation for 2 hours, cells were collected, lysed with M-PER, supplemented with 4x LDS sample buffer, boiled for 10 minutes at 95°C and used for 2D analysis. The sialyltransferase inhibitor 3F_ax_-Neu5Ac (Calbiochem, Billerica, MA) was added to 70% confluent cells at a concentration of 256 μM. Cells were collected at 24 hours after addition of inhibitor. Samples were lysed with M-PER supplemented with 4x LDS sample buffer, incubated for 10 minutes at 95°C and used for 2D analysis.

### Electrophoresis and Western blot

15 μl of the sample (10% brain homogenate) prepared as described above was loaded onto NuPAGE 12% BisTris gels (cat. # NP0341, Life Technologies, Carlsbad, CA) and run for 1 hour at 170V together with pre-stained molecular weight standard (cat # LC5925, Life Technologies, Carlsbad, Ca). Proteins were then transferred to PVDF membrane for 1 hour on ice at 33V, and PrP was detected with anti-prion protein antibody SAF-84 (epitope 160–170). Western blot signal intensity was assessed by densitometry using FluorChem imager and AlphaView software (ProteinSimple, San Jose, CA). For calculating C1/full-length PrP^C^ ratio, signal intensities of diglycosylated C1 and full-length PrP^C^ glycoforms were used. Results are presented as the mean ± Standard Deviation. Statistical significance (P) between groups were calculated by Student’s t-test, and indicated as * for significant (P<0.05) and # for insignificant (P>0.05) statistics.

### 2D electrophoresis

Samples of 25 μl volume prepared in 1xLDS were solubilized for 1h at room temperature in 200 μl solubilization buffer (8M Urea, 2% CHAPS, 5mM TBP, 20mM Tris pH 8.0), alkylated by addition of 7 μl 0.5M iodoacetamide and incubated for 1h at room temperature in darkness. Then, 1150 μl of ice-cold methanol was added, and samples were incubated for 2h at −20°C. After centrifugation at 16,000 g at 4°C, supernatant was discarded and the pellet was re-solubilized in 160 μl rehydration buffer (7M urea, 2 M thiourea, 1%DTT, 1% CHAPS, 1% Triton X-100, 0.4% ampholyte, trace amount of Bromophenol Blue). Fixed immobilized pre-cast IPG strips (cat. # ZM0018, Life Technologies) with a linear pH gradient 3–10 were rehydrated in 155 μl of the resulting mixture overnight at room temperature inside IPGRunner cassettes (cat. # ZM0008, Life Technologies). Isoelectrofocusing (first dimension separation) was performed at room temperature with rising voltage (175V for 15 minutes; 175–2,000V linear gradient for 45 minutes; 2,000V for 30 minutes) on a Life Technologies Zoom Dual Power Supply using the XCell SureLock Mini-Cell Electrophoresis System (cat. # EI0001, Life Technologies). The IPG strips were then equilibrated for 15 minutes consecutively in (i) 6 M Urea, 20% glycerol, 2% SDS, 375 mM Tris-HCl pH 8.8, 130 mM DTT and (ii) 6 M Urea, 20% glycerol, 2% SDS, 375 mM Tris-HCl pH 8.8, 135 mM iodoacetamide and loaded on 4–12% Bis-Tris ZOOM SDS-PAGE pre-cast gels (cat. # NP0330BOX, Life Technologies). For the second dimension, SDS-PAGE was performed for 1h at 170V. Immunoblotting was performed as described above. Western blots were analyzed using FluorChem M imager (ProteinSimple, San Jose, CA) and signal intensity was digitized for densitometry analysis using AlphaView software (ProteinSimple). For generating sialylation profiles, densitometry analysis of 2D blots was performed using the “Lane profile” function in the AlphaView program.

### Real-time RT-PCR

Total cellular RNA was isolated from N2a cells using Omega MicroElute Total RNA kit (Omega, Norcross, GA), according to the manufacturer's instructions. Subsequently, cDNA was synthesized from isolated RNA samples using the iScript cDNA Synthesis kit (Bio-Rad, Hercules, CA). Quantification of cDNA was done using a real-time RT-PCR machine (BioRad CFX96 Touch) and SYBR Green Fastmix (Quanta Biosciences, Gaithersburg, MD). Primers for detection of *Neu1*, *Neu3*, *Neu*, *ST3Gal3*, *ST3Gal4*, *ST3Gal6*, *ST6Gal1 and ST6Gal2* were designed using Primer-BLAST and are reported in Table 2. The relative gene expression of neuraminidases and sialyltransferases was calculated using the ΔCt method, expression in four independent repeats was analyzed for each enzyme. The housekeeping gene glyceraldehyde 3-phosphate dehydrogenase (GAPDH) was used as an internal control for normalizing relative mRNA levels in all qRT-PCR experiments.

**Table 2:** Primers for qRT-PCR (F—forward, R—reverse).

RNA Target	Sequence
GAPDH	F: 5’-CCCTCACAATTTCCATCCCAG-3’R: 5’-ATTCAAGAGAGTAGGGAGGGC-3’
NEU1	F: 5’-TCAACAGGGAAGTCTCTTCGT-3’R: 5’-AAAAGTGCTCTTGGTGACGG-3’
NEU3	F: 5’-GCTGCCCTTGATGATGGTTG-3’R: 5’-TGCAGCTAAGGACCTCTGAC-3’
NEU4	F: 5’-CCAGTTTCAGAGGATCCAACC-3’R: 5’-GGATCAAACCACCTTCAGCC-3’
ST3GAL3	F: 5’-TTGGTCCACTTGTTGTGCAG-3’R: 5’-CCTTCAGGGAGTTCACTGGT-3’
ST3GAL4	F: 5’-GTAGTCACACCGAGGCTACA-3’R: 5’-AGAGGGGATGCCACAGTAAC-3’
ST3GAL6	F: 5’-AAGTCCGTGGTGTCCATTGT-3’R: 5’-TCCAAGCATGCAGAAGGCTAT-3’
ST6GAL1	F: 5’-TACCCCAACTTTCGCTGACC-3’R: 5’-AACCATATTCCCCATGCCCT-3’
ST6GAL2	F: 5’-CACAGCGCACTGCTAAGTTC-3’R: 5’-CACAGTTAGCCCACTGGGAA-3’

### PNGase treatment

The PNGase treatment was done according to the enzyme manufacturer instructions. Briefly, 2 μl 10% brain homogenate was combined with 7 μl MilliQ water and 1 μl of 10X Glycoprotein Denaturing Buffer (included in the PNGase F set, cat # P0704S, New England Biolabs, Ipswich, MA). The glycoproteins were denatured by heating at 95°C for 5 minutes. The reaction was then chilled on ice, supplied with 2 μl 10X GlycoBuffer 2, 2 μl 10% NP40 (both reagents included in the PNGase F set), 6 μl MilliQ water and 1 μl PNGase F (cat # P0704S, New England Biolabs, Ipswich, MA). The reaction was incubated at 37°C for 1 hour, then 6.7 μl 4x SDS loading buffer was added, the samples were heated at 95°C for 10 minutes and loaded onto gel and subsequently analyzed by Western blot. SAF-84 antibody was used for staining.

### Dot-blot with PNA and β-actin

N2a lysates of cells treated or non-treated with sialyltransferase inhibitor 3F_ax_-Neu5Ac were serially diluted 2-fold in PBS. Nitrocellulose membranes (cat # 162–0115, Bio-Rad, Hercules, CA) were cut allowing for a 10x10mm^2^ area for each dot. 5 μl of each sample was applied to the membrane, which was subsequently left to dry at room temperature and then blocked in 1% BSA in PBST for 1 hour. After discarding the blocking solution, the membranes were incubated in either 8 μg/ml biotinylated peanut agglutinin or PNA (cat # BA-2301-1, EY Laboratories, San Mateo, CA) in PBST, or 1:5,000 anti-β-actin antibody (cat # A5441, Sigma-Aldrich, St. Louis, MO) in PBST for 1h at 4°C with agitation. The membranes were then washed in PBST twice for 15 minutes and incubated in the secondary antibody– 1:10,000 HRP-conjugated Streptavidin (cat # N100, ThermoFisher, Halethorpe, MD) or 1:10,000 Goat-Anti-Mouse (cat # 474–1806, KPL, Gaithersburg, MD), respectively, at room temperature. After three 10-minute washes in PBST, the membranes were developed with the Luminata Forte Western HRP substrate (cat #WBLUF500, EMD Millipore, Billerica, MA). Dot blot signal intensity was assessed by densitometry using AlphaView software (ProteinSimple). For each sample, signal intensities from PNA lectin blot were normalized by respective β-actin signal. Results are presented as the mean ± Standard Deviation.

### Treatment with A.ureafaciens sialidase

40 ul of 10% brain homogenate was mixed with 2 ul *Arthrobacter ureafaciens* sialidase (cat # P0722L, New England Biolabs, Ipswich, MA) and 10x buffer supplied with the enzyme and incubated for 5 h at 37°C in a shaker. Confluent N2a cells were collected in PBS, then Triton X-100 was added to a final concentration of 1% vol/vol and samples were s onicated for 30 seconds. 40 ul of cell lysate was mixed with 2 ul *Arthrobacter ureafaciens* sialidase (cat # P0722L, New England Biolabs, Ipswich, MA) and 10x buffer supplied with the enzyme and incubated for 5 h at 37°C in a shaker.

## Results

### Distribution of glycoforms of full-length PrP^C^, C1 and C2 fragments on 2D blots

**Fig 1:**
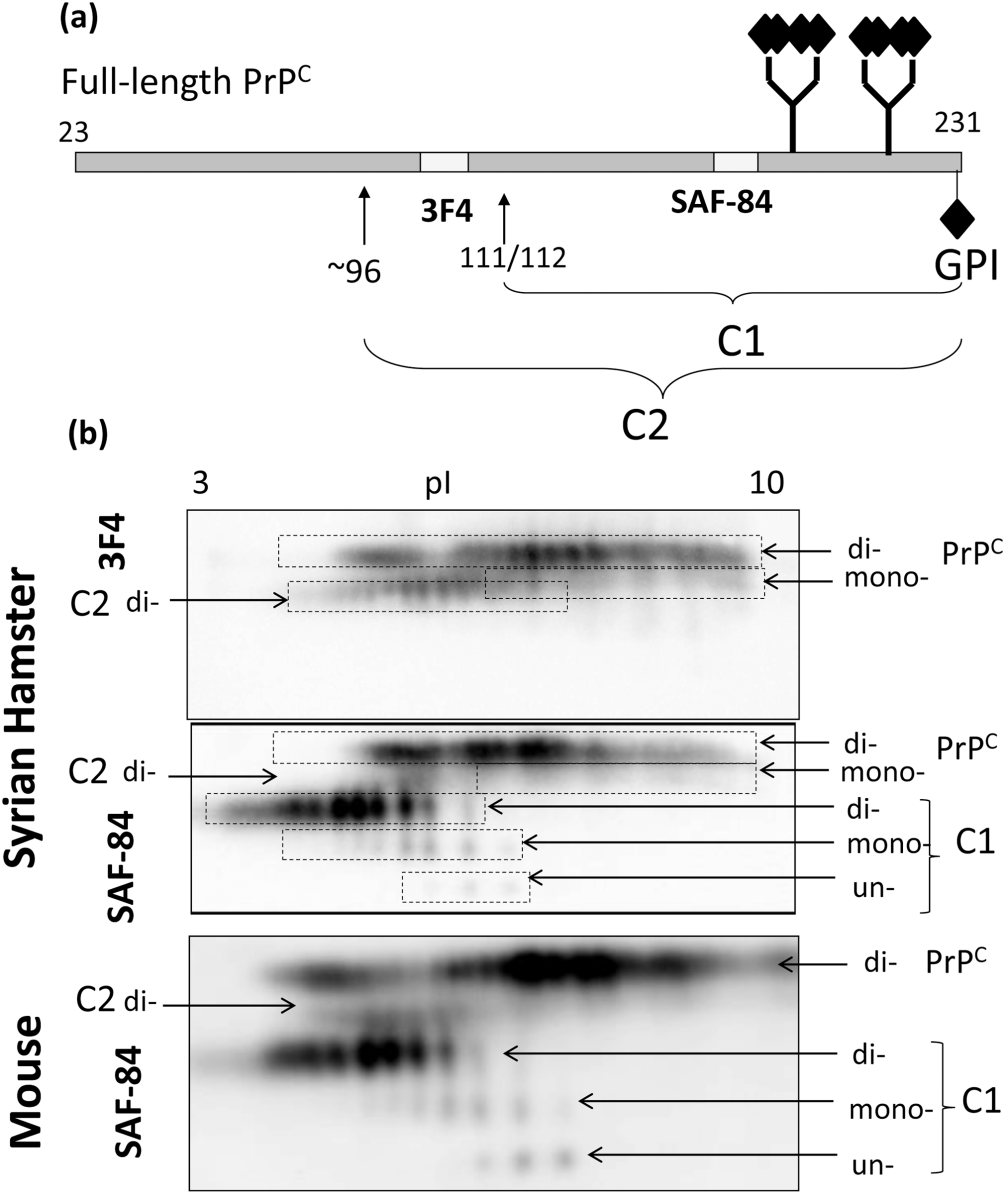
Distribution of glycoforms of full-length PrP^C^, C1 and C2 fragments on 2D blots. (a) Schematic diagram showing full-length PrP^C^ and the sites of alternative cleavage that generate C-terminal fragments C1 or C2. Positions of epitopes for 3F4 and SAF-84 antibody relative to the cleavage sites are indicated. Sialic acid residues on the N-linked glycans and the GPI-anchor are shown by a diamond symbol. (b) 2D blot analysis of brain homogenate from Syrian hamster stained with 3F4 or SAF-84 antibody, or brain homogenate from wild type mice stained with SAF-84 antibody. Syrian hamster brain material was used to map location of full-length PrP^C^, C1 and C2 fragments on 2D because antibodies toward N-terminal region of mouse PrP gave poor resolution.

**Fig 2:**
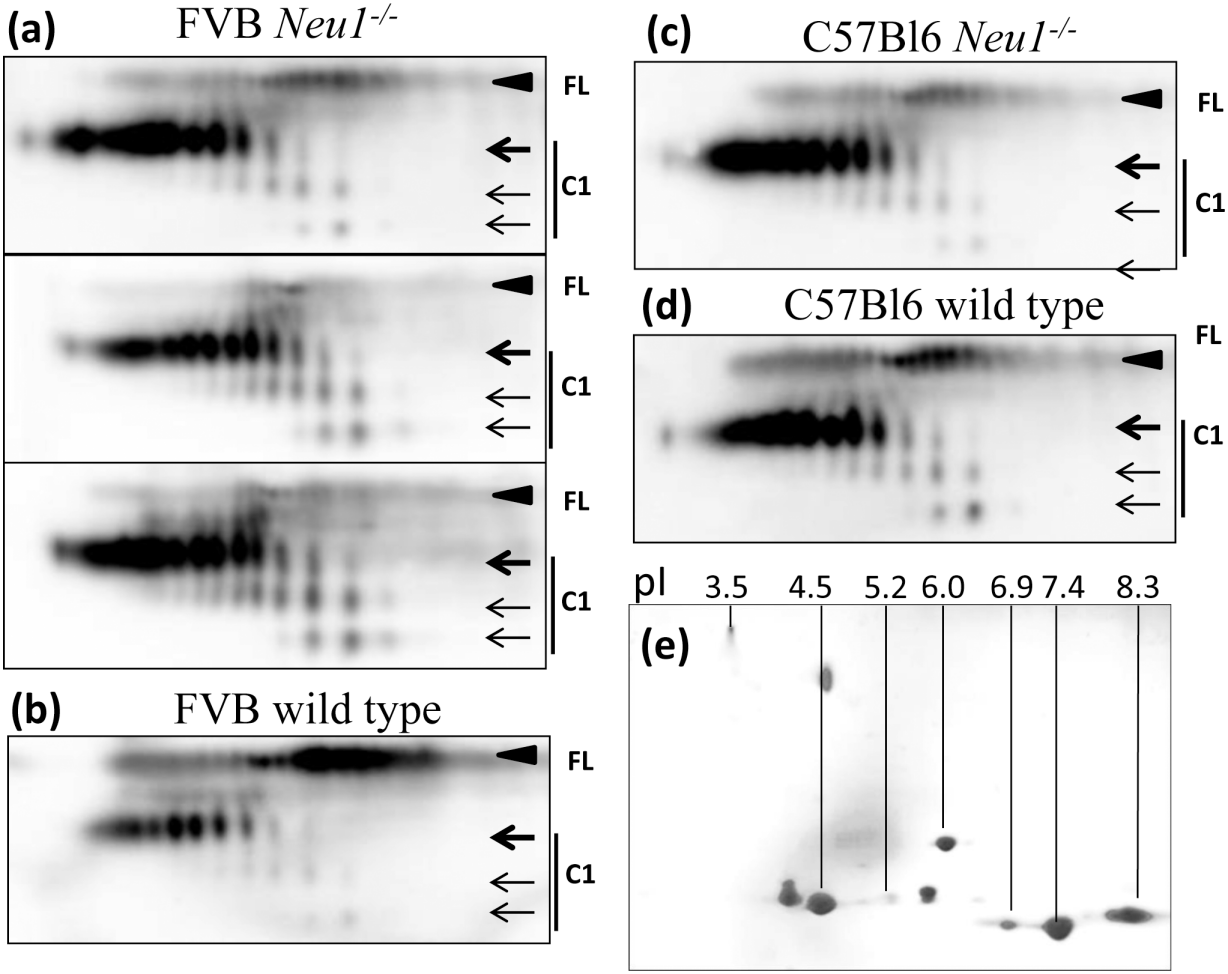
2D analysis of PrP^C^ and C1 in *Neu1* ^*-/-*^ mice. 2D Western blotting of brain homogenates from FVB *Neu1* ^*-/-*^ (a) and corresponding FVB wild type control mice (b), or C57BL6 *Neu1* ^*-/-*^ (c) and corresponding C57BL6 wild type control mice (d). 2D blots of three individual brains are shown in panel (a). 2D analysis of protein markers is provided as a reference in panel e. Filled black arrowheads mark diglycosylated full-length PrP^C^ (FL), whereas three arrows mark di-, mono- and unglycosylated forms of C1. Blots were stained with SAF-84 antibody.

In addition to full-length PrP^C^, two alternative PrP^C^-derived C-terminal proteolytic fragments C1 and C2 could be found in brain-derived materials (Fig 1). C1 is formed by a cleavage at amino acid residues 111/112, whereas C2 is produced by a cleavage near residue 96 [29, 35, 36] (Fig 1a). Like full-length PrP^C^, C1 and C2 fragments are attached to the plasma membrane via a GPI anchor and conserve two N-linked glycans [28, 29, 36]. Because each of the two N-linked glycans could contain up to four and, possibly, even five negatively charged sialic acid residues [7], the populations of diglycosylated full-length PrP^C^, C1 and C2 are expected to be highly heterogeneous with respect to their pIs. For this reason, the sialylation pattern was analyzed using 2D PAGE with a wide range of pH, 3–10. In the absence of posttranslational modifications, the theoretical isoelectric point of full-length PrP^C^ is predicted to be 9.56; however, consistent with expectations the 2D analysis revealed a wide charge distribution of full-length PrP^C^ molecules (Figs 1 and 2). As shown in our previous study [8], broad charge heterogeneity is attributed to a large extent to heterogeneity in the sialylation levels of individual PrP^C^ molecules.

**Fig 3:**
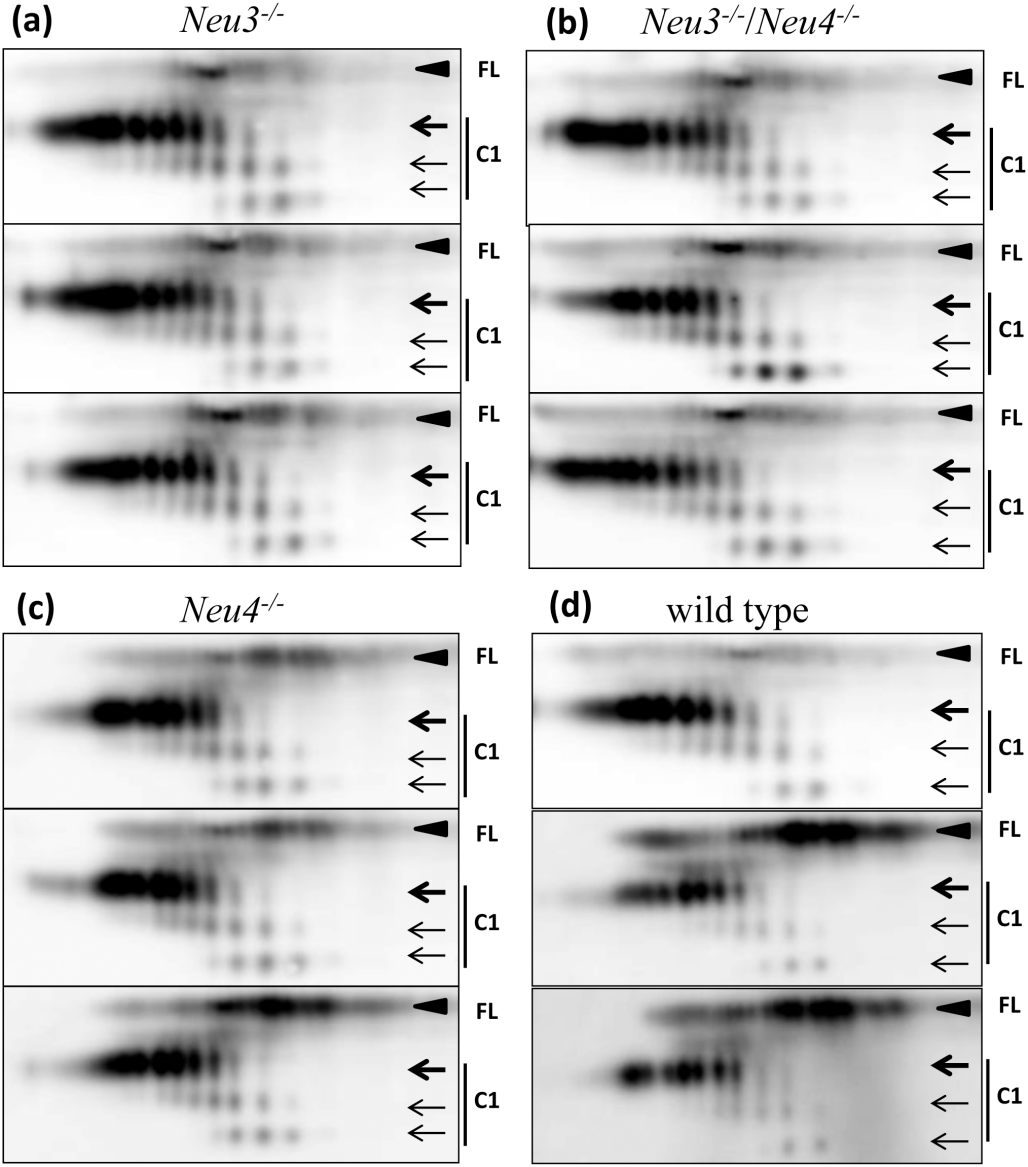
2D analysis of PrP^C^ and C1 in *Neu3* ^*-/-*^, *Neu4* ^*-/-*^ and *Neu3* ^*-/-*^/*Neu4* ^*-/-*^ mice. 2D Western blotting of brain homogenates from *Neu3* ^*-/-*^ (a), *Neu3* ^*-/-*^/*Neu4* ^*-/-*^ (b), *Neu4* ^*-/-*^ (c) and C57Bl6 wild type control mice (d). 2D blots of three individual brains are shown for each group. Filled black arrowheads mark diglycosylated full-length PrP^C^ (FL), whereas three arrows mark di-, mono- and unglycosylated forms of C1. Blots were stained with SAF-84 antibody.

Upon cleavage of the N-terminal regions, one or two positively charged amino acid clusters are removed in C2 or C1, respectively (Fig 1a). As a result, the charge of the C2 isoforms was shifted toward the acidic region relative to those of the full-length PrP^C^ on 2D gels (Fig 1b), whereas the charge of the C1 cluster was shifted even further toward the acidic region relative to those of the full-length PrP^C^ and C1 (Fig 1b). Both C1 and C2 fragments react with the C-terminal antibody SAF-84 (Fig 1a), however, C2 was barely visible as it present in very small quantities relative to that of C1 or full-length PrP^C^ (Fig 1). As expected, C1 was not detectible by antibody reactive to the epitope N-terminal to the C1 cleavage site (Fig 1b). As expected, in both C1 and full-length PrP^C^, di-glycosylated glycoforms were predominant. In fact, mono- and unglycosylated glycoforms were not detectible in full-length PrP^C^ (Figs 1, 2 and 3). Consistent with previous studies [37–39], unglycosylated C1 fragments showed up to four well-resolved charge isoforms, arguing that variation in the sialyaltion levels of the glycans was not the only source of charge heterogeneity (Figs 1, 2 and 3). Appearance of more than one charge isoforms for unglycosylated C1 is attributed to a structural heterogeneity of the GPI anchor including presence or absence of sialic acid residue on the GPI [3].

### Loss of mammalian sialidases does not influence sialylation levels of PrP^C^

Out of four known mammalian sialidases, NEU1, NEU3 and NEU4 were found to be expressed in the CNS and to localize to lysosomes and at the cell surface. These three sialidases were chosen as potential targets in regulating PrP^C^ sialylation. In addition to full-length PrP^C^, multiple charge isoforms corresponding to di-, mono-, and unglycosylated C1 proteolytic fragments were always present in substantial amounts in all brains examined (Figs 2 and 3). A shift in the distribution of charged isoforms toward an acidic pI is expected in the brains of knockout mice, if PrP^C^ is the substrate of sialidases. Surprisingly, 2D analysis of *Neu1*, *Neu3*, *Neu4* knockout brains and *Neu3/Neu4* double knockout brains revealed no notable differences in distribution of PrP^C^ charged isoforms when compared to the corresponding age-matched controls (Figs 2 and 3). The charge isoforms of C1 fragments were resolved much better than those of full-length PrP^C^; but again, they did not show any significant changes in charge distribution when compared to age-matched controls (Figs 2 and 3). Careful comparison of normalized density profiles of C1 fragments from *Neu1*, *Neu3*, *Neu4* and *Neu3/Neu4* and wild type control groups confirmed that the distributions of C1 charge isoforms were very similar in all groups (S1 Fig). As a reference, density profile of wild type brain treated with *A*.*ureafaciens* sialidase exhibited substantial shift of C1 charge isoforms toward basic pH as expected (S1 Fig). Unexpectedly, these 2D electrophoresis experiments revealed that deficiency of the sialidases expressed in the CNS does not influence the sialylation status of PrP^C^ or its proteolytic fragment C1. In 2D blots, variability in the C1/full-length PrP^C^ ratio could be observed even within the same groups (Fig 3c and 3d). Such variability was likely due to complex, multi-step experimental procedure of sample preparation for 2D, during which full-length PrP^C^ might partially precipitate. Nevertheless, despite variability the C1/full-length PrP ratio appears to increase in the *Neu1* knockout mouse brains as compared to age-matched controls (Fig 2a–2d).

### Loss of NEU1 sialidase results in increased amounts of C1 proteolytic fragment

**Fig 4:**
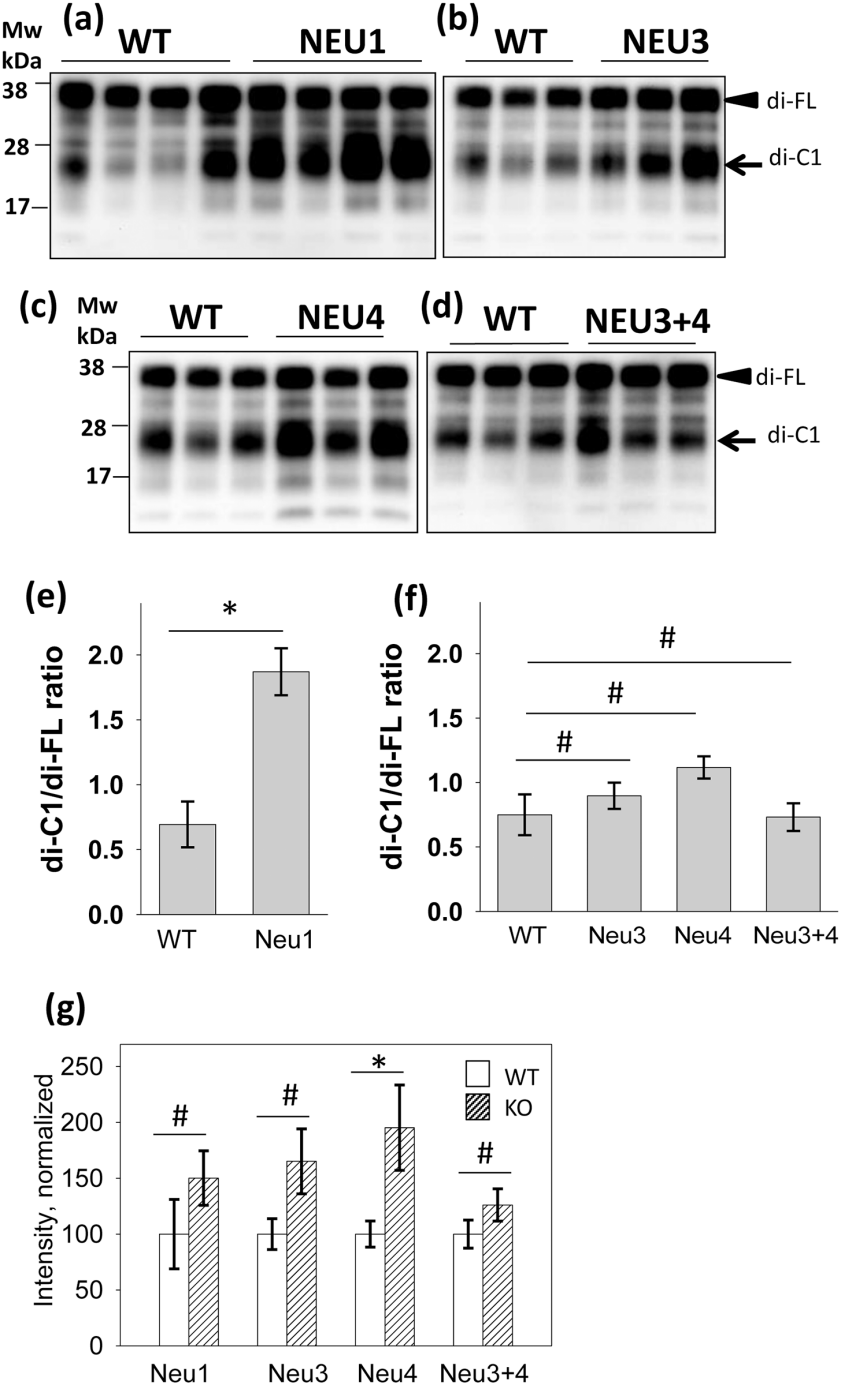
Relationship between C1 fragments and full-length PrP^C^. SDS-PAGE followed by Western blotting of brain materials from *Neu1* ^*-/-*^ (a), *Neu3* ^*-/-*^ (b), *Neu4* ^*-/-*^ (c), *Neu3* ^*-/-*^/*Neu4* ^*-/-*^ (d) and corresponding wild type controls for each group of knockout mice. Replicas within each animal groups correspond to separate mouse brains. Filled black arrowheads mark diglycosylated full-length PrP^C^ (FL), whereas arrows mark diglycosylated C1. (e, f) Ratio of diglycosylated C1/ dyglycosylated full-length PrP^C^ from densitometry analysis for FVB *Neu1* ^*-/-*^ and corresponding FVB wild type mice (e), or C57BL6 *Neu3* ^*-/-*^, *Neu4* ^*-/-*^, *Neu3* ^*-/-*^/*Neu4* ^*-/-*^ and corresponding C57BL6 wild type mice (f) is plotted for each animal group. (g) The total amounts of both full-length PrP^C^ and C1 fragments in *Neu1* ^*-/-*^, *Neu3* ^*-/-*^, *Neu4* ^*-/-*^ and *Neu3* ^*-/-*^/*Neu4* ^*-/-*^ and corresponding wild type controls. The signal intensity in each group is normalized relative to the intensity of corresponding wild type controls. The data represent means ± SD from three mouse brains for each group.* indicate significant differences (P<0.05, n = 3), whereas # indicate lack of significant differences (P>0.05, n = 3).

To document the changes in the C1/full-length PrP^C^ ratio, brain materials from *Neu’s* knockout mice were analyzed using SDS-PAGE (Fig 4a–4d). The C1/full-length PrP^C^ ratio was found to be 2-fold higher in *Neu1* knockout brains than in control animals or in *Neu3*, *Neu4* or in the *Neu3/Neu4* double knockout brains (Fig 4e and 4f). Treatment with PNGase confirmed that full-length PrP^C^ and C1 were the main PrP material in the brain homogenates (S2 Fig). Western blots stained with antibody Ab3551 (epitope 90–102) failed to detect C1 fragments (S2 Fig), providing an additional support that the major band marked as di-C1 corresponds to diglycosylated C1 fragment (Fig 4a–4d). Notably, an increase in the C1/full-length PrP^C^ ratio was attributable mostly to higher levels of C1 fragments in *Neu1* knockout brains relative to the controls (Fig 4). In addition to a high C1/full-length PrP^C^ ratio in *Neu1* knockout brains, all lines of knockout mice appear to show a tendency for higher amount of total PrP signal (C1 plus full-length PrP^C^) in comparison to the corresponding wild type controls (Fig 4g). Although, only in *Neu4* knockout brains this difference was statistically significant.

### Inhibition of sialidases does not change sialylation pattern in N2a cells

**Fig 5:**
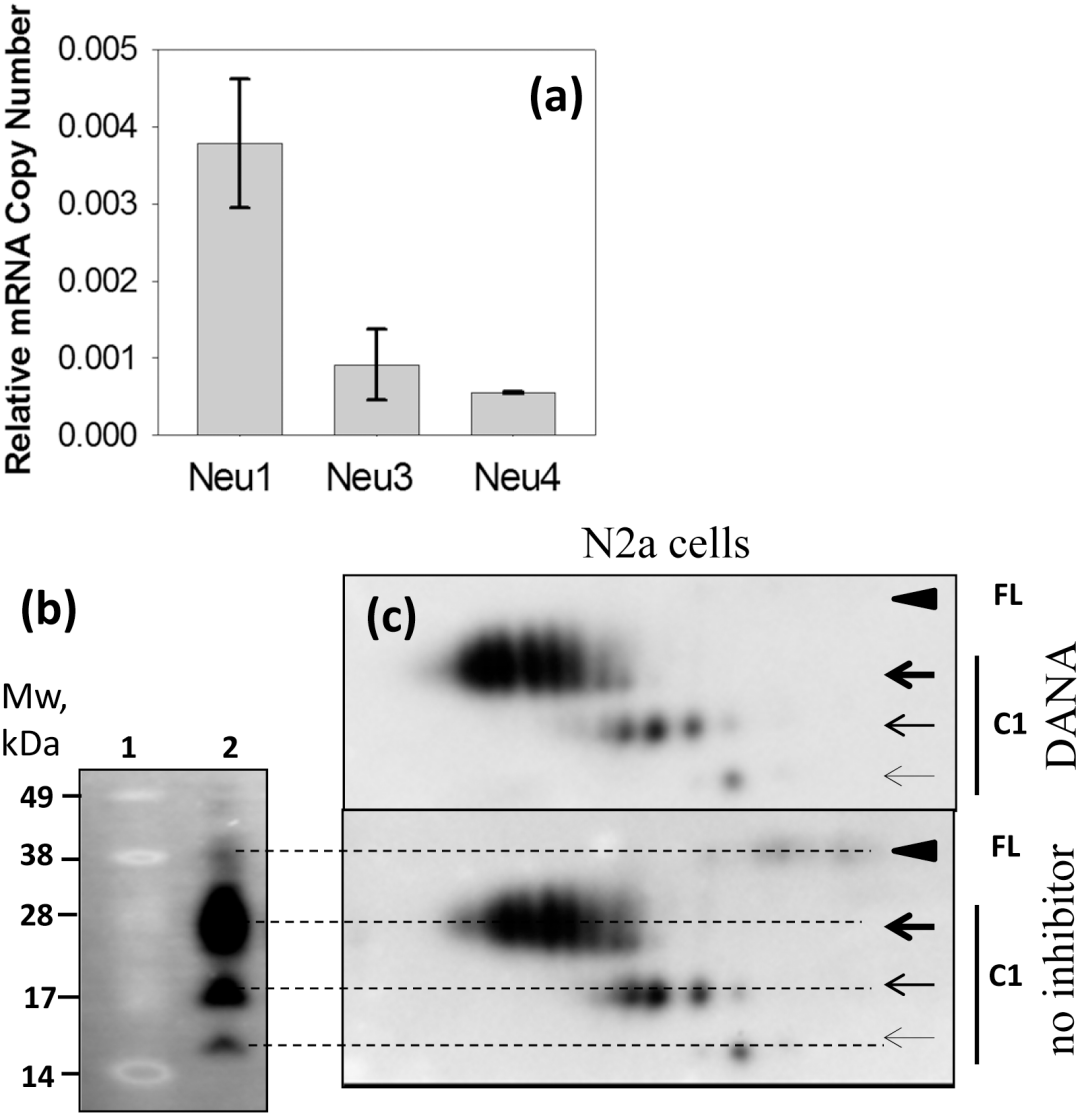
Inhibition of sialidases does not alter sialylation patterns in N2a cells. (a) The mRNA levels for Neu1, Neu3, and Neu4 in N2a cells were normalized to the glyceraldehyde 3-phosphate dehydrogenase internal control. Mean +/- Standard Deviation (n = 4) are shown. (b) Western blotting of N2a cell lysate (lane 2) demonstrating large amounts of the C1 proteolytic fragment. Molecular weight markers are shown on lane 1. (c) 2D Western blotting of cell lysate of N2a cells cultured in the absence of neuraminidase inhibitor (lower panel) or in the presence of 5 mM DANA for 2 hours (upper panel). Filled black arrowheads mark diglycosylated full-length PrP^C^ (FL), whereas three arrows mark di-, mono- and unglycosylated forms of C1. Blots were stained with SAF-84 antibody. Three independent experiments were performed.

As an alternative approach for testing whether PrP^C^ sialylation status could be altered by changing the levels of cellular NEUs, neuroblastoma N2a cells were treated with DANA (2,3-didehydro-2-deoxy-*N*-acetylneuraminic acid) (Fig 5). DANA is a general, nonselective inhibitor of mammalian and viral neuraminidases with IC_50_ values for mammalian NEUs in micromolar range [40]. Real-time PCR confirmed expression of *Neu1*, *Neu3* and *Neu4* mRNAs in N2a cells. The relative levels of *Neu3* and *Neu4* mRNAs was approximately 4–5 fold lower than that of *Neu1* resembling the expression rank order in the brain (Fig 5a, Table 2). While N2a cell line has been extensively used in prion research for replicating prions in cultured cells, the majority of full-length PrP^C^ in N2a is processed proteolytically yielding large amounts of the C1 fragment (Fig 5b and 5c). For this reason, we used the charge distribution of C1 fragments to report on sialylation status in N2a cells. Despite the high concentration of DANA (5 mM) applied to the cells, we could not detect any changes in the sialylation pattern towards negative pI (Fig 5b). The absence of any effects of DANA further reinforced the notion that targeting sialidases is not an effective strategy for altering the sialylation status of PrP^C^.

### Inhibition of sialyltransferases shifts sialylated isoforms distribution in N2a cells

**Fig 6:**
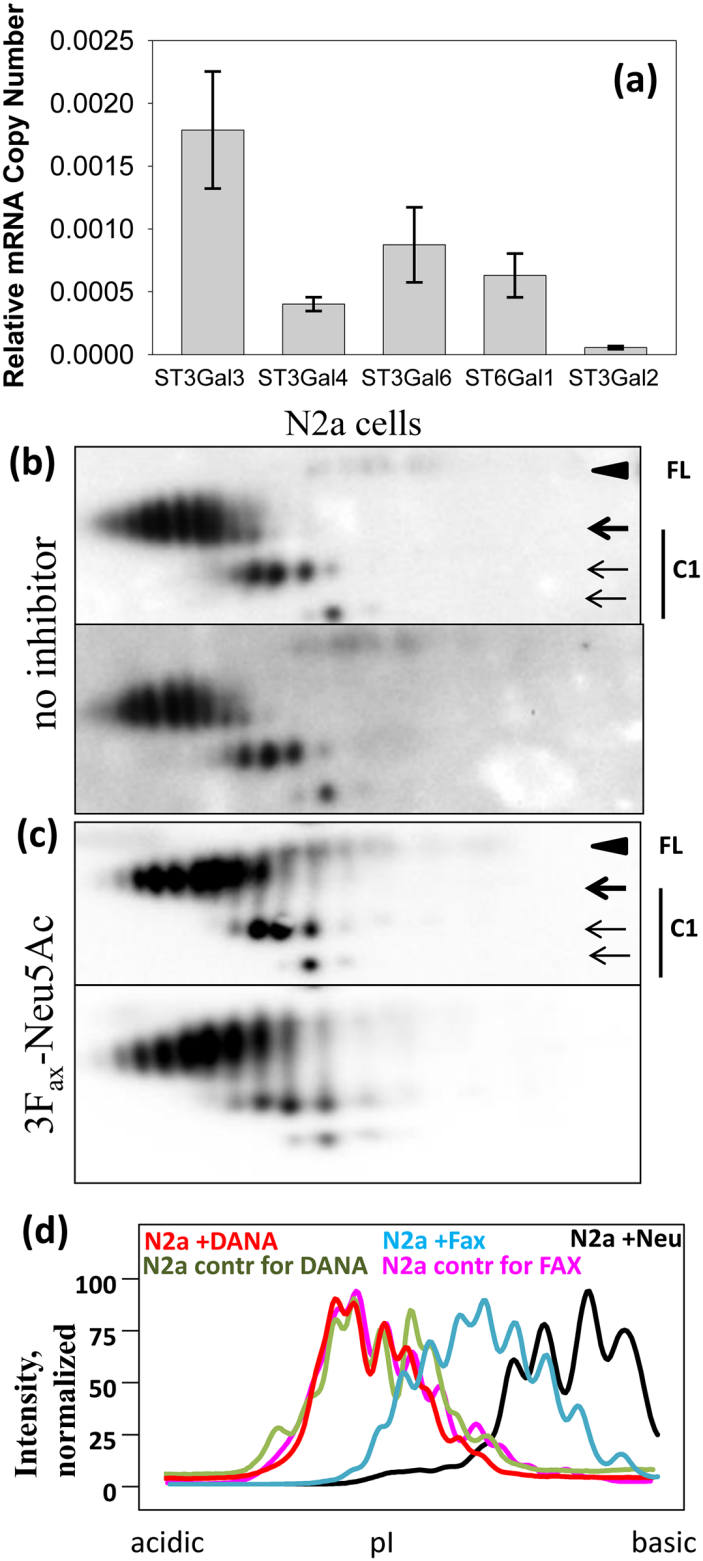
Inhibition of sialyltransferases alters sialylation pattern in N2a cells. (a) The mRNA levels for ST3Gal3, ST3Gal4, ST3Gal6, ST6Gal1 and ST6Gal2 in N2a cells were normalized to the glyceraldehyde 3-phosphate dehydrogenase internal control. Mean +/- Standard Deviation (n = 4) are shown. (b,c) 2D Western blotting of cell lysate of N2a cells cultured in the absence of sialyltransferase inhibitor (b) or in the presence of 256 μM 3F_ax_-Neu5Ac for 24 hours (c). Filled black arrowheads mark diglycosylated full-length PrP^C^ (FL), whereas three arrows mark di-, mono- and unglycosylated forms of C1. Blots were stained with SAF-84 antibody. The experiment was performed twice with two independent repeats each time. 2D blots from each independent experiment are shown. (d) Sialylation profiles of diglycosylated C1 fragment isoforms for N2a cells cultured with 3F_ax_-Neu5Ac (Fax, blue) and corresponding control cells (pink), or N2a cells cultured with DANA (red) and corresponding control cells (green). Sialylation profile of N2a cells treated with *A*.*ureafaciens* sialidase (Neu, black) is provided as a reference.

To test whether the distribution of sialylated isoforms could be changed by inhibiting the activity of sialyltransferases, we employed a general inhibitor of these enzymes, peracetylated 3F_ax_-Neu5Ac [41, 42]. Upon cellular uptake this compound is transformed into CMP-3F_ax_-Neu5Ac. While CMP-3F_ax_-Neu5Ac is a poor substrate for sialylatransferase, it can inhibit the enzymes efficiently [41, 42]. Accumulation of CMP-3F_ax_-Neu5Ac in a cell leads to depletion of a natural substrate of sialylatransferases, CMP-NeuAc, via a metabolic feedback loop [11]. Out of twenty sialylatransferases, the following five enzymes are responsible for α2->3 or α2->6 sialylation of N-linked glycans: ST3Gal3, ST3Gal4, ST3Gal6, ST6Gal1 and ST6Gal2. Real-time PCR revealed that N2a cells expressed all five enzymes, although at different levels (Fig 6a). N2a cells cultured in the presence of 3F_ax_-Neu5Ac showed substantial increase in binding of peanut agglutinin PNA, a lectin that specifically recognize Gal-β(1–3)-GalNAc carbohydrate sequence in asialoglycans (S3 Fig). This experiment confirmed that treatment of cells with 3Fax-Neu5Ac significantly reduces bulk sialylation level of total glycans. C1 fragments from N2a cells cultured in the presence of 3F_ax_-Neu5Ac exhibited a shift in charge distribution towards positive pI (Fig 6b and 6c). As expected, a right shift was observed for diglycosylated and, to a lesser extent, monoglycosylated glycoforms of the C1 fragment. Comparison of normalized density profiles of C1 diglycosylated isoforms confirmed that treatment with 3F_ax_-Neu5Ac leads to a shift in charge distribution towards basic pI (Fig 6d). In a control experiment, N2a cells treated with *A*.*ureafaciens* sialidase exhibited more substantial shift of C1 charge isoforms toward basic pH than cells cultured with 3F_ax_-Neu5Ac illustrating that inhibition of sialyltransferase activity by 3F_ax_-Neu5Ac was partial (Fig 6d). Notably, cells cultured in the presence of DANA did not show any shift in the distribution of C1 charge isoforms (Fig 6d). This result illustrates that targeting sialyltransferases rather than sialidases may offer a more effective strategy for modulating the PrP^C^ sialylation status.

## Discussion

Our earlier studies revealed a correlation between sialylation status of PrP^C^ and the propensity of PrP^C^ to engage in replication of PrP^Sc^ *in vitro*. Enzymatically desialylated PrP^C^ was found to exhibit a much higher conversion rate in PMCAb than PrP^C^ with unaltered sialylation status [8]. Surprisingly, animals inoculated intracerebrally with PMCAb-derived 263K material generated from de-sialylated PrP^C^ lacked any clinical signs of prion disease or prion-associated pathology [8]. The brains and spleens of dsPMCAb-inoculated hamsters were PrP^Sc^-negative on Western blotting. Notably, PrP^Sc^ amplified in PMCAb in normal brain homogenate was found to be less sialylated and showed a longer incubation time to disease relative to brain-derived PrP^Sc^. These results document that a correlation between PrP^Sc^ sialylation status and incubation time to disease exists [8]. While desialylated PrP^C^ increases the rate of prion replication *in vitro*, the resulting PrP^Sc^ lacks infectivity or ability to propagate in animal brain and the lymphoreticular system. Combined, these data argue that the sialylation status of both PrP^C^ and PrP^Sc^ are of paramount importance to prion pathogenesis. Consequently, it may be of great interest to explore the possibilities for modulating prion protein sialylation *in vivo* and its potential implication for the development of new therapeutic approaches.

PrP^C^ synthesis and trafficking have been subjects of a number of studies (reviewed in [43]). After synthesis in the ER, PrP^C^ undergoes posttranslational modifications including glycosylation, sialylation and attachment of the GPI-anchor in the Golgi. The synthesis and transport of PrP^C^ to the cell surface is estimated to take approximately one hour [44, 45]. As judged from pulse-chase experiments, the half-life of PrP^C^ in N2a cells was 5 hours [44], whereas the half-life in mouse-derived primary cerebellar cells or splenocytes was found to be only 1.5–2 hours [45]. This discrepancy suggests that *in vivo* PrP^C^ might be degraded much faster than in cultured cells. PrP^C^ cycles between the cell surface and endocytic compartments, where a minor fraction of PrP^C^ is degraded, while the major fraction of the protein returns intact to the cell surface [35, 46, 47]. In addition, PrP^C^ undergoes proteolytic cleavage at two alternative sites referred to as α- or β-cleavage that yields the C-terminal C1 or C2 fragments, respectively [29, 35, 36]. The half-life of C1 or C2 fragments is not known. A minor amounts of PrP^C^ is released from a membrane by a metalloprotease-mediated cleavage between amino acid residues 228 and 229 that chop off the GPI-anchor [48, 49].

**Fig 7:**
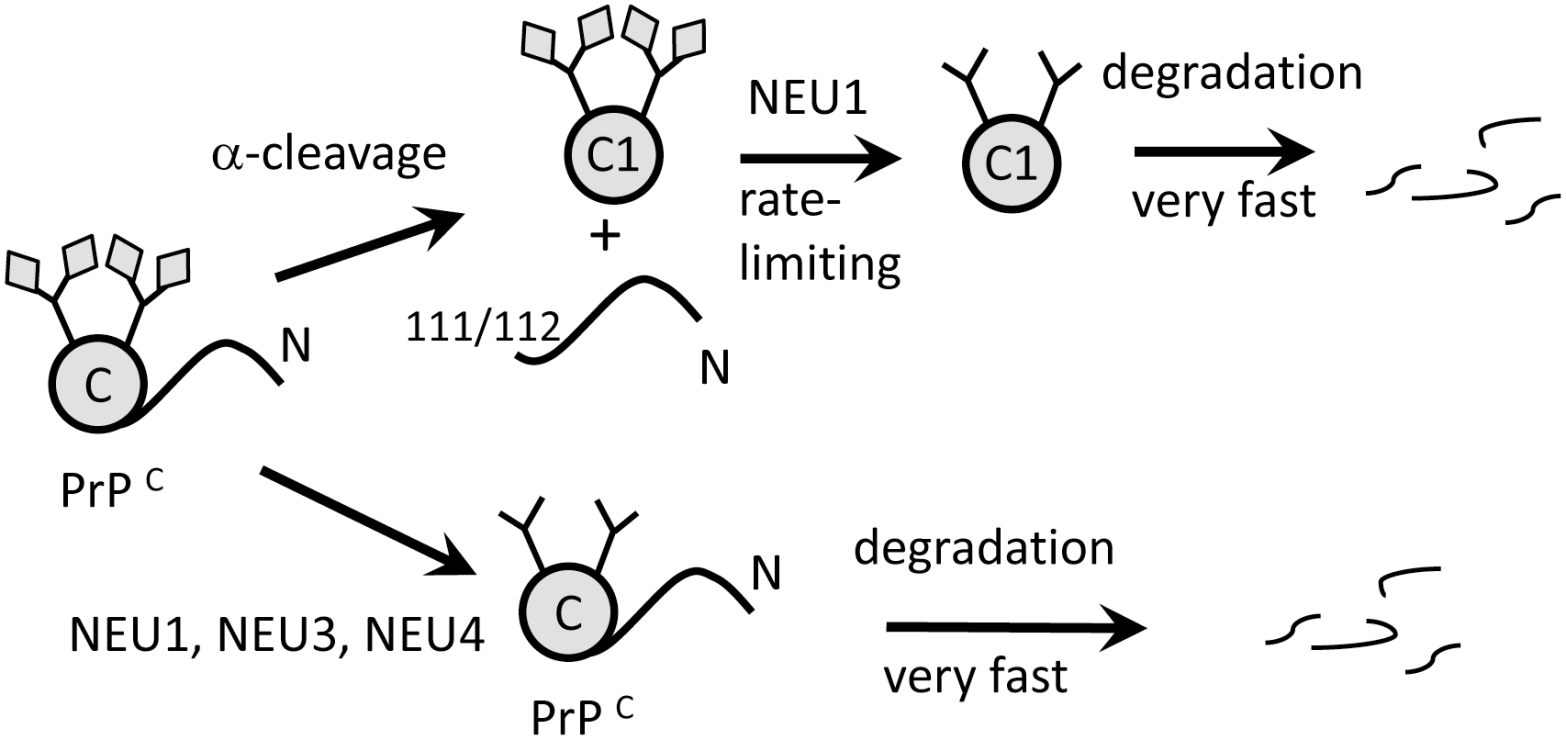
Hypothetical mechanism of PrP^C^ degradation. Two alternative degradation pathways are proposed. The first pathway involves α-cleavage as a first step, which produces C1 fragment. Following α-cleavage, the C1 fragment undergoes desialylation by NEU1, a process that triggers its very fast degradation. The model predicts that desialylation of C1 is required for its degradation, therefore NEU1 deficiency leads to accumulation of C1. According to the second pathway, full-length PrP^C^ is desialylated directly in the absence of α-cleavage. The C-terminal folded domain and C1 are depicted as circles; sialic acid residues are shown as diamonds.

Because PrP^C^ is localized at the cell surface and in endocytic/lysosomal compartments, NEU1, NEU3 and NEU4 were the enzymes selected for this study based on their tissue expression and cellular localization (Table 1). Brain materials from mice deficient in NEU1, NEU3, NEU4 or NEU3 and NEU4 were analyzed using 2D Western blots. Surprisingly, the sialylation status of PrP^C^ or its proteolytic fragment C1 did not change in any of the *Neu’s* knockouts. Moreover, inhibition of cellular sialidases with the general sialidase inhibitor DANA did not alter the sialylation status of the C1 proteolytic fragment in N2a cells despite its very high concentration of 5 mM (Fig 5). DANA is a general, nonselective inhibitor of mammalian neuraminidases with IC_50_ values 76, 6.3 and 13 μM for NEU1, NEU3 and NEU4, respectively [40]. At 1 mM DANA was found to suppress dramatically total neuraminidase activity in rat brain slices [50], whereas no change in sialylation status of C1 was observed in the current study at 5 mM DANA. It is possible that PrP^C^/C1 molecules are degraded so fast following desialylation that the relative contribution of desialylated PrP^C^/C1 in the total pool is too small to be detected (Fig 7). If fast degradation is indeed triggered by desialylation, sialidase deficiency should lead to accumulation of PrP^C^ and/or its proteolytic fragment C1..Indeed, higher amounts of total PrP signal (C1 plus full-length PrP^C^) were observed in *Neu1*, *Neu3*, *Neu4* knockout brains relative to the corresponding wild type controls, although, statistically significant difference was seen only in *Neu4* knockout group (Fig 4g). Cellular localizations of NEU1, NEU3, NEU4 overlap partially, therefore, it is likely that these three NEUs act on different cellular sub-populations of PrP^C^/C1. We propose that NEU1 is responsible for desialylation of C1 fragment glycans, and that desialylation triggers rapid degradation of C1 (Fig 7). This hypothesis predicts that in the absence of NEU1, C1 accumulates because sialylation protects C1 against degradation. When NEU1 is expressed, desialylation of C1 is followed by its very rapid degradation. Rapid degradation of C1 would keep the population of desialylated C1 so low that its contribution to sialylation profile of total C1 would not be detectible. Consistent with this proposed mechanism, the change in C1 to full-length PrP^C^ ratio in *Neu1* knockout mice was primarily due to increased amounts of C1, whereas the amount of full-length PrP^C^ remained constant. Establishing turn-over rates for C1 and PrP^C^ experimentally in future studies would help to test this hypothesis.

The data on subcellular sites of α-cleavage remain controversial (reviewed in [51]). Harris and co-authors proposed that α-cleavage occurs in lysosomes, as inhibitors of lysosomal proteases were found to block the cleavage [35]. Hooper and coauthors detected C1 at the cell surface and produced several lines of evidence that α-cleavage occurs in a late compartment of the secretory pathway [52]. The identity of proteolytic enzymes responsible for α-cleavage has been controversial as well. Different classes of proteolytic enzymes, including ADAM9, ADAM10, ADAM17 [53, 54], ADAM8 [55] and plasmin [56, 57], have been implicated in α-cleavage. However, subsequent studies challenged some of these findings [49, 58, 59]. The current work reconciles some of the differences regarding cellular sites of α-cleavage. In a previous study NEU1 was shown to suppress lysosomal exocytosis, a process by which lysosomal proteolytic enzymes are excreted into the extracellular space [60]. In particular, *Neu1* knockout mice were found to display an increase in lysosomal exocytosis and extracellular proteolytic activity [60]. Elevated extracellular proteolytic activity explains well the substantial increase in the ratio of C1 to full-length PrP^C^. Taken together, the current work supports the view that α-cleavage might involve proteases of lysosomal origin.

NEU1 was found to be important in controlling the ratio of full-length PrP^C^ to its proteolytic fragment C1 (Fig 4). The C1/full-length PrP^C^ ratio is important because it contributes to the rate of disease progression and determines incubation time to disease [61]. Not only is C1 unable to serve as a substrate for PrP^Sc^ replication, but this fragment was also found to inhibit PrP^Sc^ replication in a dominant-negative manner [61]. In mouse the ratio of C1 to full-length PrP^C^ appears to vary in different brain regions, where C1 was found to be most abundant in the cerebellum [62]. In the current study, the ratio of C1 to full-length PrP^C^ was found to vary even between animals within the same group (Fig 4). Such variability might be because several alternative mechanisms are involved in α-cleavage that might take place in several cellular sites. While it would be interesting to test whether prion disease is delayed in *Neu1* knockout mice, these mice, unfortunately, do not have a sufficient lifespan, as they suffer from sialidosis, muscle degeneration and Alzheimer’s disease-like amyloidogenic processing of an oversialylated amyloid precursor protein in lysosomes [60, 63, 64].

The current study demonstrated that targeting cellular sialidases does not provide an effective strategy for manipulating the sialylation status of full-length PrP^C^. In the absence of detectable changes in PrP^C^ sialylation status in *Neu’s* knockout models, manipulating sialylation status via sialyltransferases represented the next logical step. In mammals, there are at least twenty sialytransferases (STs) that form four groups according to the type of carbohydrate linkages they synthesize and substrate specificity [42, 65]. STs are localized in the Golgi, where they are responsible for transferring sialic acid residues from CMP-NeuAc to the terminal positions of glycoproteins or glycolipids [10, 42]. The peracetylated 3F_ax_-Neu5Ac used in this study is a general inhibitor that targets a broad spectrum of STs [41, 42]. While full-length PrP^C^ was barely detectable on 2D Western blot, the distribution of C1 charge isoforms showed a clear shift towards the positive pI upon incubation with the inhibitor. The shift in charge distribution is indicative of a reduction in the sialylation level of PrP glycosyls. Recently, 3F_ax_-Neu5Ac was shown to be effective in suppressing global sialylation when administered to mice [66]. However, its use in animals led to irreversible kidney dysfunction and death emphasizing the needs for developing more specific inhibitors of sialyltransferases [66]. STs of two families ST3Gal and ST6Gal are responsible for attaching sialic acids to N-linked glycans at the positions α2->3 or α2->6, respectively, the type of sialylation found in PrP^C^ [6, 7]. There are eight STs within these two families. Identification of those STs that are in charge of PrP^C^ sialylation will be the subject of future studies.

## Supporting Information

S1 Fig Sialylation profiles of diglycosylated C1 fragment isoforms in brain materials of *Neu1* ^*-/-*^ (black lines), *Neu3* ^*-/-*^ (red lines), *Neu3* ^*-/-*^/*Neu4* ^*-/-*^ (green lines), *Neu4* ^*-/-*^ (blue lines) and wild type controls (gray lines). Brain materials from three independent animals within each group were analyzed. Brain material from wild type animal treated with *A*.*ureafaciens* sialidase (dotted lines) is provided as a reference.
(PDF)

S2 Fig **(a)** PNGase treatment of brain materials from *Neu1* ^*-/-*^ and wild type control mice. Brain materials from *Neu1* ^*-/-*^ and corresponding wild type control mice were treated with PNGase, analyzed by Western blot and stained with SAF-84 antibody. PNGase treatment confirmed that di-glycosylated full -length PrP^C^ and C1 are two major forms present in the brain material. **(b)** Western blots of brain materials from *Neu1* ^*-/-*^ and wild type control mice stained with Ab3531 and SAF-84 antibodies. Filled black arrowheads mark diglycosylated full-length PrP^C^ (FL), whereas arrows mark diglycosylated C1.
(PDF)

S3 Fig Lectin binding assay. **(a)** N2a cells were cultured in the presence of absence of 3F_ax_-Neu5Ac as described in Fig 6, lysed and the amounts of asialoglyans were quantified by dot blot stained with lectin PNA. Three independent cell culture replicas are shown for each group. Two-fold serial dilutions of cell lysates were loaded to illustrate dose dependence. Dot blot of β -actin was perform as an internal control. **(b)** Quantitative analysis of the data presented in panel (a). Signal intensities of PNA staining were normalizes per intensities of corresponding β -actin.
(PDF)
