# Supplementary figures and images for: Loss of Cellular Sialidases Does Not Affect the Sialylation Status of the Prion Protein but Increases the Amounts of Its Proteolytic Fragment C1
Source: PLoS One. 2015 Nov 16;10(11):e0143218. doi: 10.1371/journal.pone.0143218 (PMC4646690; doi:10.1371/journal.pone.0143218)

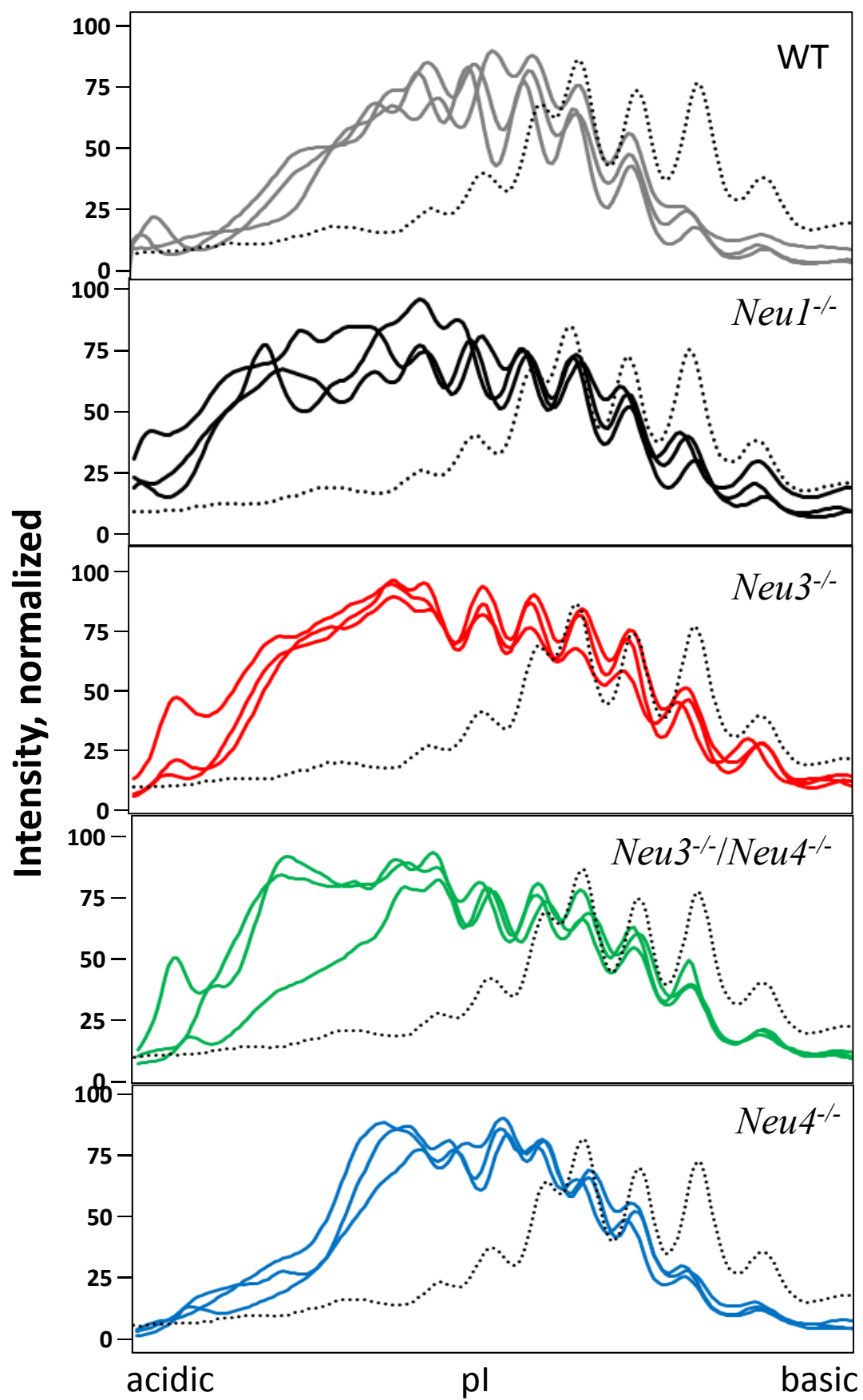

**Figure S1**

Supplement: S1 Fig — Brain materials from three independent animals within each group were analyzed. Brain material from wild type animal treated with A.ureafaciens sialidase (dotted lines) is provided as a reference. (PDF) [file pone.0143218.s001.pdf]

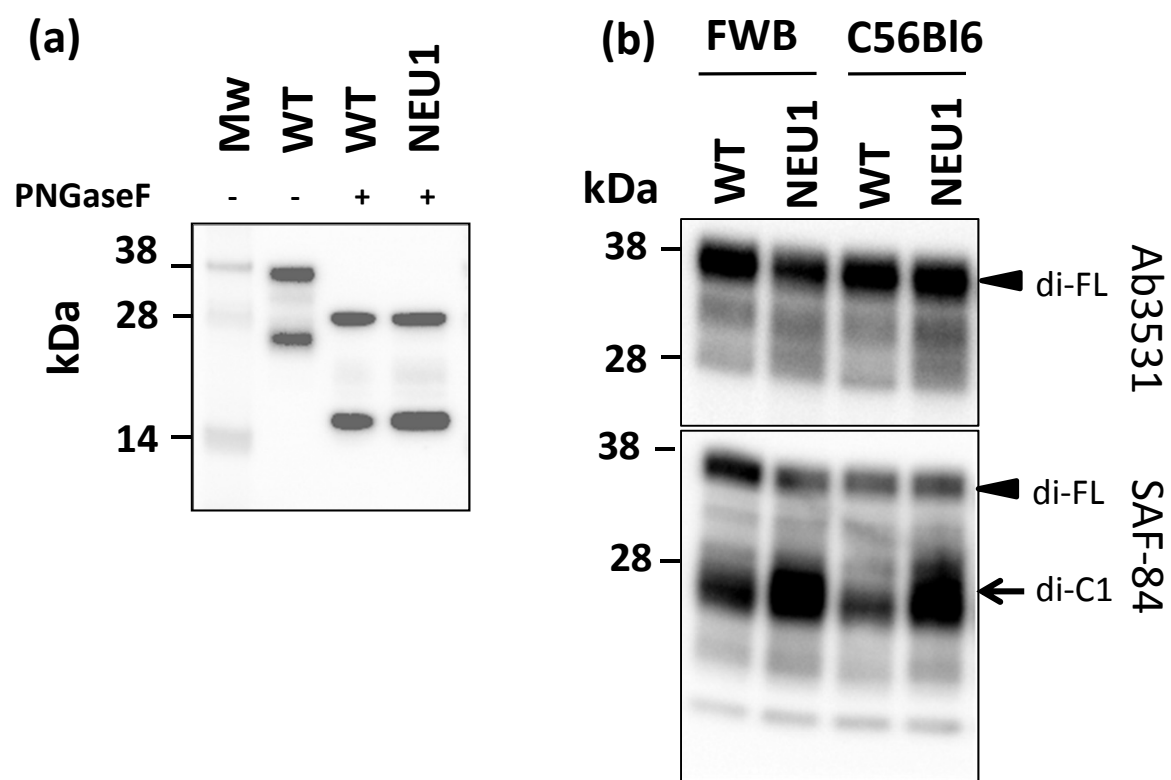

Figure S2

Supplement: S2 Fig — (a) PNGase treatment of brain materials from Neu1 -/- and wild type control mice. Brain materials from Neu1 -/- and corresponding wild type control mice were treated with PNGase, analyzed by Western blot and stained with SAF-84 antibody. PNGase treatment confirmed that di-glycosylated full -length PrPC and C1 are two major forms present in the brain material. (b) Western blots of brain materials from Neu1 -/- and wild type control mice stained with Ab3531 and SAF-84 antibodies. Filled black arrowheads mark diglycosylated full-length PrPC (FL), whereas arrows mark diglycosylated C1. (PDF) [file pone.0143218.s002.pdf]

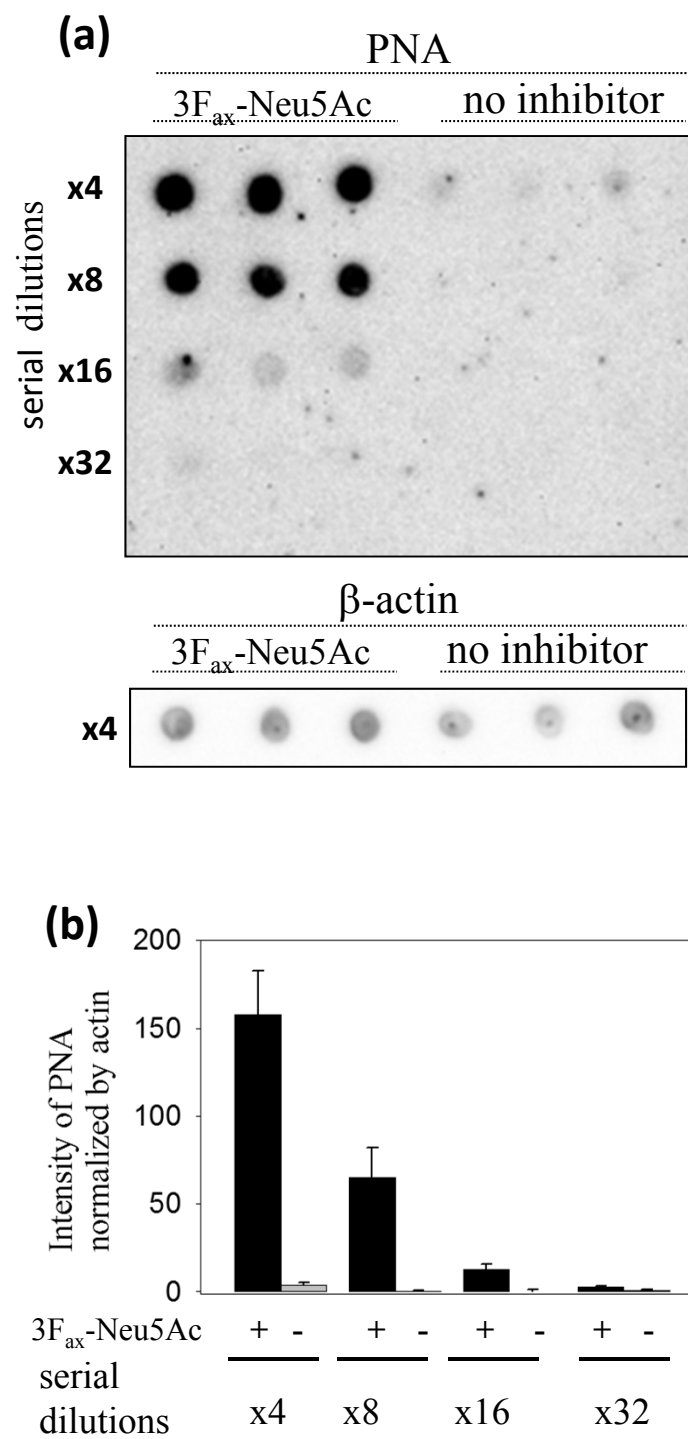

**Figure S3**

Supplement: S3 Fig — (a) N2a cells were cultured in the presence of absence of 3Fax-Neu5Ac as described in Fig 6, lysed and the amounts of asialoglyans were quantified by dot blot stained with lectin PNA. Three independent cell culture replicas are shown for each group. Two-fold serial dilutions of cell lysates were loaded to illustrate dose dependence. Dot blot of β -actin was perform as an internal control. (b) Quantitative analysis of the data presented in panel (a). Signal intensities of PNA staining were normalizes per intensities of corresponding β -actin. (PDF) [file pone.0143218.s003.pdf]
